# Case Report: Human Dermatitis Linked to *Ornithonyssus bursa* (Dermanyssoidea: Macronyssidae) Infestation in Portugal

**DOI:** 10.3389/fvets.2020.567902

**Published:** 2020-12-14

**Authors:** Helga Waap, Dora Aguin-Pombo, Maria Maia

**Affiliations:** ^1^Laboratório de Parasitologia, Instituto Nacional de Investigação Agrária e Veterinária, Oeiras, Portugal; ^2^CIISA, Faculdade de Medicina Veterinária, Universidade de Lisboa, Lisboa, Portugal; ^3^Faculty of Life Science, University of Madeira, Funchal, Portugal; ^4^CIBIO, Centro de Investigação em Biodiversidade e Recursos Genéticos, Universidade do Porto, Vairão, Portugal; ^5^Fundação para a Ciência e Tecnologia, Lisboa, Portugal

**Keywords:** *Ornithonyssus bursa*, avian-mite dermatitis, bird-mite dermatitis, gamasoidosis, chickens, poultry, Portugal

## Abstract

*Ornithonyssus bursa* (Berlese, 1888), also known as the tropical fowl mite, is a blood-feeding ectoparasite of domestic and wild birds. It is considered a serious pest to poultry in warm and tropical climates and has been reported to attack humans, causing gamasoidosis. Avian-mite dermatitis may be difficult to recognize and misdiagnosed as bites from other arthropods. The present report describes two cases of human dermatitis linked to *O. bursa* infestation. Both cases occurred in an apartment in a residential area in Oeiras, Portugal, where two members of the same family presented with pruritic erythematous skin eruptions disseminated over their body over a period of 4 months. The lesions were located mostly in the pelvic, gluteal, coccygeal, and perineal regions, and also on the neck, wrists and torso. On inspecting the mattresses and the covers of the bedrooms, three mites ~1 mm long by 0.5 mm wide were found. The three specimens were identified as *O. bursa*. Investigations tracing back the source of infestation, revealed that the mites were carried over from chickens raised 45 km away (Sesimbra, Setúbal) that, after being slaughtered for consumption, had been transported by car to the apartment in Oeiras. The chickens were farmed in an open backyard henhouse that allowed free access to several species of wild birds, including pigeons (*Columba livia*), turtle doves (*Streptopelia decaocto*), and sparrows (*Passer domesticus*). Recent reports suggest that *O. bursa* may be established in Mediterranean countries, increasing the risk of gamasoidosis. This is the first record linking *O. bursa* infestation of chickens with human dermatitis in continental Portugal. More research is needed to assess the extent of poultry infestation and evaluate the possible implications for the poultry industry, as well as for human health in Portugal.

## Introduction

The Dermanyssoidea (*Acari, Mesostigmata)* superfamily comprises most of the mite parasites of vertebrates, including haemathophagous species in the Dermanyssidae and Macronyssidae families, some of which are considered zoonotic (1). Among them, non-burrowing avian mites occur such as *Dermanyssus gallinae* (De Geer, 1778; Dermanyssidae) also known as the poultry red mite, and *Ornithonyssus (O.) sylviarum* (Canestrini and Fanzago, 1877) and *O. bursa* (Berlese, 1888; Macronyssidae), known as the northern and tropical fowl mite, respectively. These dermanyssoid bird mite species naturally parasitize both wild nesting birds and domestic fowl and they also are of veterinary and medical concerns. In fact, in the absence of the natural host, these mites may occasionally feed on humans, causing avian mite dermatitis. Cutaneous manifestations are characterized by pruritic, erythematous, or urticarial papules, often presenting a central sting (2). Due to their small size, about 1 mm in length, and the habit to leave the human skin after feeding (3), infestations may go unnoticed and avian mite dermatitis be misdiagnosed as other skin conditions. In Europe, bird mite attacks are reported among people working with poultry (4) but the incidence of gamasoidosis is increasing in urban settings due to the close proximity of nests of synanthropic birds (2, 4). While *D. gallinae* has a worldwide distribution, *O. sylviarum* is mostly linked to temperate regions and *O. bursa* to tropical and subtropical territories, though recent records suggest it may be also established in Southern Europe. To the best of our knowledge, to date no records for *O. bursa* were available for continental Portugal. The aim of the present report is to describe for the first time a case of avian mite dermatitis linked to *O. bursa* infestation of chickens in continental Portugal.

## Case Description

**Figure 1:**
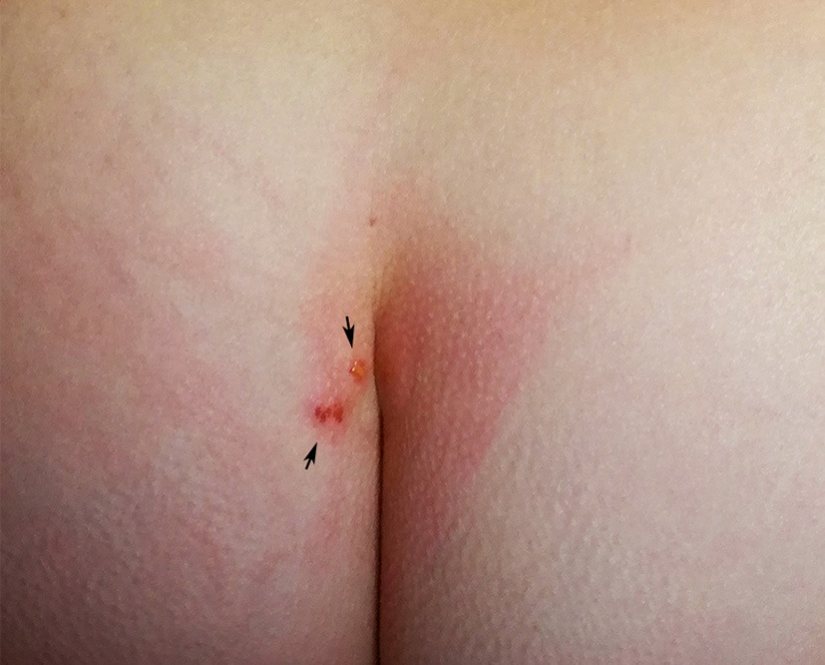
Skin lesions due to *Ornithonyssus bursa* (arrows).

A 51 years old woman contacted the parasitology laboratory of INIAV (Instituto National de Investigação Agrária e Veterinária) for advice on a possible bird mite infestation. The woman reported that she and her 16 years old daughter, who lived in the same apartment, located in the residential area of Oeiras, had been suffering from pruritic erythematous papules of unknown origin, which occurred continuously, with more or less intensity, over a period of 4 months (February-June 2019). The lesions were located primarily in the pelvic, gluteal, coccygeal (Figure 1), and perineal regions, under the breast, and to a lesser extent on the neck, wrists and torso. The intense pruritus and consequent scratching of some papules resulted in wide inflamed skin areas. Lesions were particularly intense and painful around the waist. Bothers occurred habitually at night, but were also noticed during daytime, in which case the waistline was the most affected area. Pruritus was described as severe and prolonged in time and exacerbated by increasing temperature, including by contact with hot water, e.g., while showering or washing the dishes. An initial search in the apartment had not revealed any arthropods visible to the naked eye. After some months, in which no source of dermatitis was found, the mother, who had gained experience in the field of veterinary parasitology during her studies, hypothesized that the cutaneous reactions could be caused by bird mites, which, due to their small size could have gone unnoticed. During an interview it was turned out that the affected individuals raised chickens for egg consumption in an open backyard henhouse in their weekend residence in Sesimbra (Setúbal), 45 km away from Oeiras. The owners used to enter the henhouse weekly to collect eggs; direct contact with chickens occurred merely when hens were captured to be slaughtered for consumption. Thereafter, the chickens were usually transported by car to the apartment in Oeiras. No protective clothing was worn during these activities. The henhouse was accessible to several species of wild and synanthropic birds, including pigeons (*Columba livia*), turtle doves (*Streptopelia decaocto*) and sparrows (*Passer domesticus*). Upon questioning, the women also remembered seeing mites crawling on her hands when plucking the chickens. No other sources of mite infestation could be identified, e.g., the presence of active or abandoned bird nests near windows or pets living in the apartment (cats, dogs or birds). Based on the clinical and epidemiological features, it was suspected that dermatitis was caused by avian mites conveyed by chickens from the farm to the apartment. The residents were advised to thoroughly inspect all environments and hiding places suitable for bird mites in the apartment, e.g., beddings, frequently used furniture (desks, tables, couch), cracks and crevices, skirting boards, door and window frames. On inspecting the beddings, the mother retrieved three mites of ~1 mm on the linens of the bed where she had slept previously. The specimens were sent to INIAV for morphological identification.

**Figure 2:**
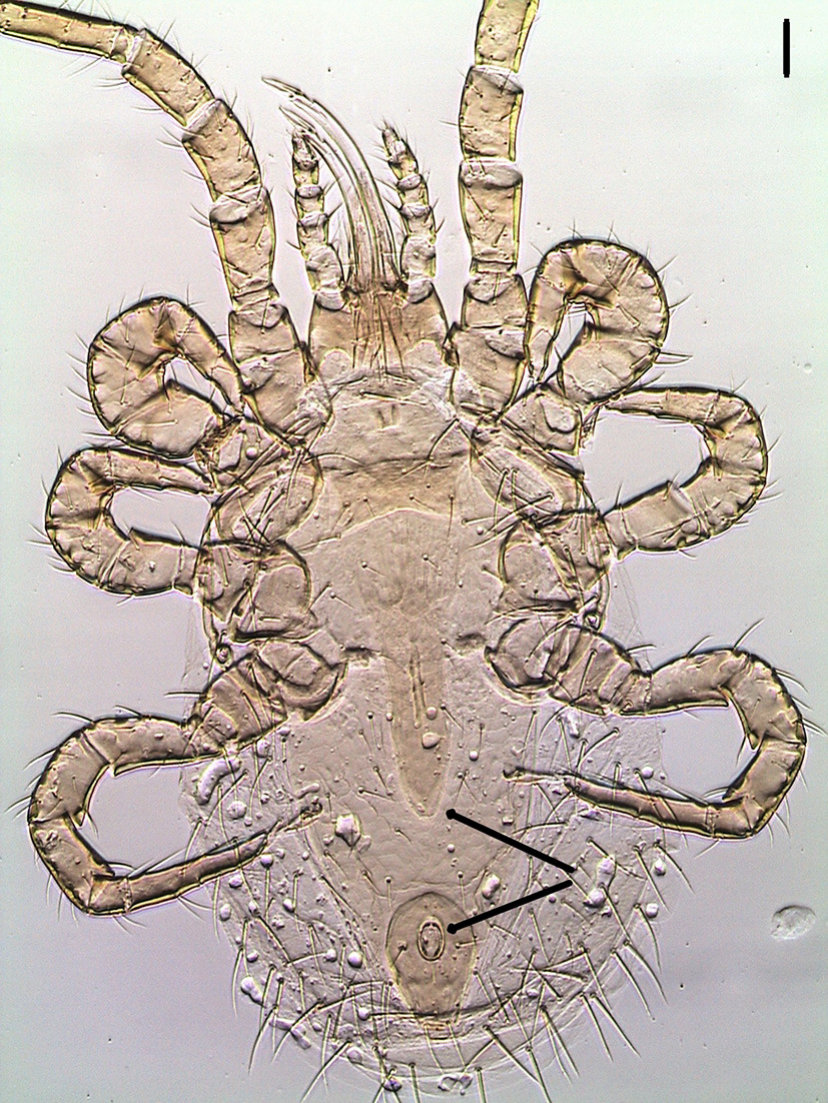
Ventral view of *Ornithonyssus bursa* adult female showing the posteriorly narrowed genitoventral shield (upper arrow) and the teardrop-shaped anal plate with anal opening at the anterior end (lower arrow). Scale bar: 50 μm.

**Figure 3:**
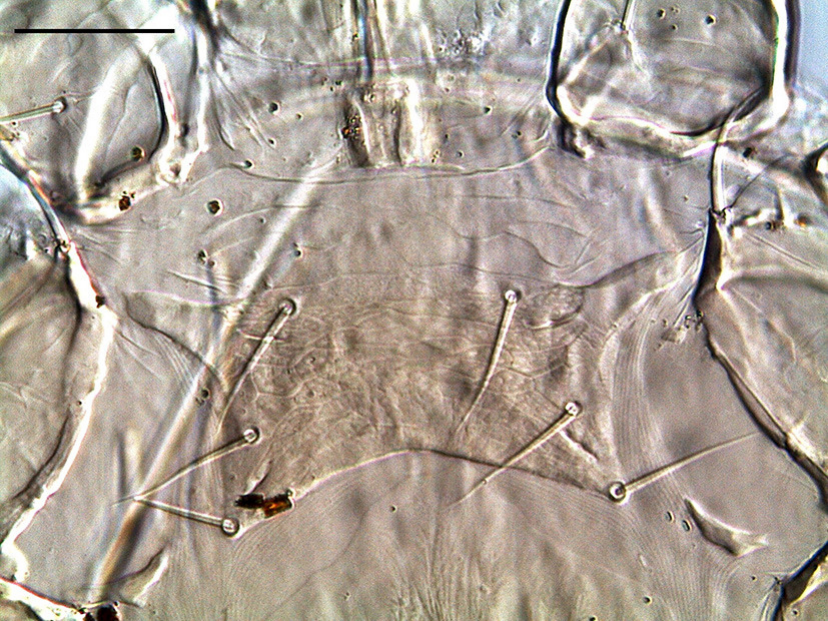
Detail of the sternal shield bearing three pairs of setae. Scale bar: 50 μm.

Mites were cleared with potassium hydroxide 10%, mounted on microscope slides with Hoyer's medium and identified under a Leica Microsystems™ DM IL LED inverted microscope equipped with phase contrast and integrated modulation contrast (IMC) optics. Photomicrographs were taken with a Leica Microsystems™EC3 digital camera using IMC. The three mites were identified as *O. bursa* females based on the following morphological characteristics: tritosternum present, a posteriorly narrowed genitoventral shield, dorsum of body with relatively few setae, elongate, edentate chelicerae, sternal plate wider than long, short, shearlike chelae with well-developed, distinct fixed and movable digits, a teardrop shaped anal plate with anal opening at the anterior end (Figure 2), single holodorsal shield gradually decreasing in width posteriorly; a j6 setal pair shorter, not reaching the bases of following pair and the third pair of setae clearly on posterior corners of sternal shield (5–8) (Figure 3).

After the identification of *O. bursa*, the woman was asked to search the henhouse in Sesimbra for potential mites, in order to confirm the source of infestation. In addition, she was asked to place cardboard traps 7 x 10 cm in size, commonly used to capture *D. gallinae* (9) near perches and nests. Mites were collected with help of a fine forceps and magnifying glass and stored in 70% alcohol. Traps were frozen at −20° during 24 h to inactivate mites before shipment to INIAV. A total of 17 *O. bursa* and one *D. gallinae* specimens were collected. Most of the *O. bursa* mites were retrieved on perches, which were made of tree-branches, thus providing a perfect place for mites to live and hide near chickens, while the only *D. gallinae* specimens was found in one of the cardboard traps.

Cutaneous manifestations in mother and daughter resolved without medical intervention within 20 days after removal of mites from the apartment. This was achieved by intensive vacuum cleaning, washing of clothes and bedlinen with laundry detergent and hot water (60°C) and thorough daily inspection of mattresses, clothing and bedding. In order to avoid relapses, it was recommended to avoid the contact with infested birds.

## Discussion

Gamasoidosis by avian mites is currently an increasing but neglected global problem (4). The causative agents *D. gallinae, O. sylvarium* and *O. bursa* are spread worldwide. These mites naturally infect an extensive range of avian hosts, including poultry and several species of wild, pet, and synanthropic birds. Known primarily as pests of poultry, the importance of the three mite species has traditionally been ascribed to different geographical regions and climates. *D. gallinae* is considered the most important ectoparasite for the laying industry, particularly in Europe, where it causes major economic losses to 80% of poultry farms (10). Likewise, *D. gallinae* is highly prevalent in Portugal, with over 90% of layer farms affected (11). *O. sylviarum* occurs throughout the temperate regions of the world, but is primarily important in North America, where it is regarded as the most common and damaging ectoparasite of poultry (12), Brazil (13, 14), China (15), and Australia (16). In Europe, *O. sylviarum* was the most frequent mite in nests of wild birds in Slovakia, Italy and Austria (17–19). It was occasionally also found in poultry (20), pheasants and canaries in France (21), in ornamental chicken breeds in Sweden (22) and, more recently also in pet birds in Portugal (23), but infestation levels are by far not comparable with those of *D. gallinae* (24). The reasons for the different infestation patterns between continents remain unclear (21). *O. bursa*, the least studied of these mites, is considered to be almost entirely restricted to warm and tropical regions. Its presence has been reported in domestic fowl and wild birds in several countries in South America, Africa, Australia, and Asia and islands of the Caribbean Sea, Indian, and Pacific Ocean (6, 25–31). In Europe, its avian hosts include swallows in Denmark (32), rock pigeons and monk parakeets in Spain (33) and backyard chickens in the Madeira Island, Portugal (34).

Human injuries are caused by mites that migrate from abandoned nests of synantrophic birds, like sparrows, starlings, doves and feral pigeons, to residences (2). These bird species are very common in urban areas and usually build their nests on facades of buildings, roofs, windows, chimneys, behind air conditioning units, under eaves, or attics (3, 4, 35). When nests are abandoned, food-seeking mites may intrude residential or occupational settings through ventilation ducts, cracks, and crevices near windows, ceiling and walls. In other instances, avian mites may attack humans working with severely infested poultry or when farmers, workers or visitors are not wearing adequate protective clothing, with *D. gallinae* regarded as an occupational hazard (4, 36). Mite attacks in residential areas occur typically during the night, as opposed to occupational cases, which occur mostly during the day (4). This was also observed in the present situation, though bites were also occasionally noticed during the day, probably due to mites carried inside clothing during working operations. Frequently, patients with bird mite dermatitis present to clinics in late spring and early summer, soon after young birds fledge and adults leave their nests. This was not the case here, because mites were carried over from backyard chickens raised all year round by the owners. Reports on urban cases of avian mite dermatitis have increased in recent years and currently outweigh the reported cases linked to poultry farming (4). *O. sylviarum* and *D. gallinae* are the mites most frequently implicated in gamasoidosis, the latter with over 170 outbreaks registered in Europe (4, 37). Although comparatively less common, human infestations by *O. bursa* have been reported before in urban residential areas of India (38) and Brazil (35, 39–41). In both cases the source of mites was traced to abandoned bird nests. Recently, isolated urban cases of *O. bursa* infestation were reported in an Italian 70-years-old male (Sicily, Palermo) (42) and in a couple (76-years-old, male and 70-years-old, female) in Spain (Girona) (43); the cause of the infestation was attributed to presence of backyard chickens and dove nests in the garden, respectively. Although in both cases mites were only collected on patients and not on birds, the papular dermatitis disappeared after removal of chickens and dove nests, confirming this as the probable source of infestation. In the herein reported case, *O. bursa* was collected and identified in the residence, as well as in the henhouse perches and nests confirming carryover of mites from the infested farm to the apartment.

Despite the increasing number of reports worldwide, gamasoidosis is still a frequently unrecognized ectoparasitosis in humans (2, 3). Diagnosis is challenging, particularly when a direct link to birds cannot be readily established. As humans are unnatural hosts, mites leave shortly after feeding and are only rarely detected on human skin. The small mites are barely visible to the naked eye and may go unnoticed for several months before the causal relationship can be established. In general, gamasoidosis tends to be temporary and self-limiting (44). However, if mites are not found and removed in the environment of the patient, there may be recurrence and exacerbation of the lesions (45). Further, diagnostic errors can occur, because skin manifestations are nonspecific. Therefore, several other ectoparasites need to be considered in the differential diagnosis, including fleas, scabies, pediculosis, baker's itch, *Cheyletiella* bites in pet owners, infestation by Trombiculidae (chiggers), or even bed bug bites (2, 45). Thus, clinical cases can only be precisely diagnosed if ectoparasites are isolated and collected. This case report shows that, although uncommon, poultry farmed in premises outside of the residence area, even if located far away, can act as a source of urban infestation by avian mites. A possible carryover of bird mites from poultry kept elsewhere should therefore be considered in the anamnesis of patients presenting cutaneous manifestations compatible with gamasoidosis.

The present report describes for the first time in Portugal two cases of human dermatitis presumably caused by *O. bursa* and it also confirms the presence of the mite in farmed chickens in continental Europe, also in co-existance with *D. gallinae*. Though a possible carryover of *D. gallinae* into the apartment cannot be completely ruled out, as one specimen was retrieved in the traps placed later in the henhouse, the finding of only *O. bursa* in the bed linens, enabling prolonged skin contact with one of the afflicted individuals, strongly suggests this mite as the cause of the observed dermatitis. Based on previous records in wild and synatrophic birds and human infestation in other Mediterranean countries, the present findings suggest that *O. bursa* can be prevalent in the Mediterranean basin but it could be overlooked or misdiagnosed (38, 39, 42). The typical Mediterranean climate is characterized by warm to hot, dry summers and mild to cool, wet winters; these conditions could be suitable for this tropical mite to develop. Another hypothesis could be a recent introduction of *O. bursa*, either by new wild bird populations or by the importation of fowl or exotic pet bird species, followed by spreading in the Old Continent (42). Irrespective of reasons for actual distribution, the short life cycle of *O. bursa* allows it to quickly build up large populations in poultry farms. Therefore, monitoring of *O. bursa* in wild and domestic avian hosts in Portugal is essential in order to evaluate and mitigate the risk of dissemination to poultry. The increasing number of avian mite attacks to city dwellers, suggest that these infestations may become an emerging public health problem in urban environments. Global warming and ecological changes may favor species range expansion of tropical birds and associated ectoparasites. Likewise, *O. bursa* may expand northward into temperate latitudes increasing the risk for gamasoidosis. More attention to this matter based on a One-Health approach is needed. This supposes a greater awareness of avian dermatitis by physicians/dermatologists and a closer collaboration with veterinarians and entomologists in order to establish a correct diagnosis and treatment, identify the sources of infestation and undertake adequate measures to prevent and control this zoonotic ectoparasite.

## Data Availability Statement

The original contributions presented in the study are included in the article/supplementary material, further inquiries can be directed to the corresponding author/s.

## Ethics Statement

Written informed consent was obtained from the individual(s) for the publication of any potentially identifiable images or data included in this article.

## Author Contributions

HW performed the morphological identification of mites and wrote the report. DA-P and MM provided critical feedback, helped shape the manuscript and contributed to the writing. MM performed a literature review and supervised the work. All authors contributed to the article and approved the submitted version.

## Conflict of Interest

The authors declare that the research was conducted in the absence of any commercial or financial relationships that could be construed as a potential conflict of interest.
